# Latent trajectories of frailty and risk prediction models among geriatric community dwellers: an interpretable machine learning perspective

**DOI:** 10.1186/s12877-022-03576-5

**Published:** 2022-11-24

**Authors:** Yafei Wu, Maoni Jia, Chaoyi Xiang, Ya Fang

**Affiliations:** 1 School of Public Health, Xiamen University, Xiang’an Nan Road, Xiang’an District, Xiamen, 361102 Fujian China; 2 National Institute for Data Science in Health and Medicine, Xiamen University, Xiamen, Fujian China; 3 Key Laboratory of Health Technology Assessment of Fujian Province, School of Public Health, Xiamen University, Xiamen, Fujian China

**Keywords:** Frailty trajectories; machine learning, Group-based trajectory modeling, SHapley additive exPlanations

## Abstract

**Background:**

This study aimed to identify long-term frailty trajectories among older adults (≥65) and construct interpretable prediction models to assess the risk of developing abnormal frailty trajectory among older adults and examine significant factors related to the progression of frailty.

**Methods:**

This study retrospectively collected data from the Chinese Longitudinal Healthy Longevity and Happy Family Study between 2002 and 2018 (*N* = 4083). Frailty was defined by the frailty index. The whole study consisted of two phases of tasks. First, group-based trajectory modeling was used to identify frailty trajectories. Second, easy-to-access epidemiological data was utilized to construct machine learning algorithms including naïve bayes, logistic regression, decision tree, support vector machine, random forest, artificial neural network, and extreme gradient boosting to predict the risk of long-term frailty trajectories. Further, Shapley additive explanations was employed to identify feature importance and open-up the black box model of machine learning to further strengthen decision makers’ trust in the model.

**Results:**

Two distinct frailty trajectories (stable-growth: 82.54%, rapid-growth: 17.46%) were identified. Compared with other algorithms, random forest performed relatively better in distinguishing the stable-growth and rapid-growth groups. Physical function including activities of daily living and instrumental activities of daily living, marital status, weight, and cognitive function were top five predictors.

**Conclusions:**

Interpretable machine learning can achieve the primary goal of risk stratification and make it more transparent in individual prediction beneficial to primary screening and tailored prevention.

**Supplementary Information:**

The online version contains supplementary material available at 10.1186/s12877-022-03576-5.

## Background

The frailty, a complex syndrome, is endorsed as “a medical syndrome with multiple causes and contributors that was characterized by diminished strength, endurance, and reduced physiologic function that increases an individual’s vulnerability for developing increased dependency and/or death” by American Federation for Aging Research [1]. Frailty is common in older adults, often accompanied by heterogeneous decline of physiologic capacity [2]. Many assessment tools were used to define frailty in screening, the frailty phenotype and the deficit accumulation approach were recognized as two operational approaches [3]. The Fried frailty phenotype consists of five physical components: unintentional weight loss, self-reported exhaustion, weakness, slow walking speed, and low physical activity, the presence of three or more of above was identified as frailer [4]. The cumulative deficit approach is to calculate a continuous score named Frailty Index (FI), a ratio of the number of deficits present in the individual to the number of total deficits, ranging from 0 (no deficit) to 1 (all deficits present) to reflect the state of frailty. FI can be derived from epidemiological survey to describe the intrinsic onset and progression of physiological decline in the aging process.

Worldwide, the evaluated prevalence of frailty was about 24%, based on the FI, among people aged over 50 years old [5]. With an aging process, the prevalence is projected to increase. Older people living with frailty have an increased risk of falls, fractures, hospitalizations, thus would lower life quality, even lead to higher risk of disability and early mortality, bringing a substantial challenge to public health [6, 7]. Therefore, it is urgent to take effective strategies for frailty management, especially pragmatic screening. There emerging a large body of studies aimed to explore for early identification of frailty and it’s worth noting that frailty was recognized as a dynamic state, existing heterogeneity in population, with different initial manifestations often leading to different trajectories of frailty progression [6]. Many modifiable risk factors such as lifestyles, medical resources, education, living condition, obesity, chronic disease, and physical inactivity were verified to be related to individual frailty trajectory [8–11]. So, it is significant to identify latent sub-populations of frailty trajectories, and examine their possible risk factors, which would be promising to realize tailored interventions, consistent with the advocation of precision prevention, treatment and management. However, in practice, it is still overlooked to predict who will develop frailty more likely, whatever in the community or clinic [12]. With the rapid development of computer science, machine learning (ML) gradually comes into public insight, playing a crucial role in decision-making in all fields. It can deal with multidimensional data to give predictions about individuals beneficial to diagnosis, prognosis, and treatment in clinic settings, proved superior to traditional methods [13, 14]. As we all know, things always go like a spiral, new challenges emerging in the progression from the maturity of algorithm to practical use. One of them is model interpretability, encountering with distrust and limited model application [15].

The study attempted to address the following issues: first, group-based trajectory modeling (GBTM) was used to describe individual heterogeneous frailty trajectories over a 16-year period among the 65+ adults of the Chinese Longitudinal Healthy Longevity and Happy Family Study (CLHLS-HF). Second, we gave prediction of frailty trajectory with ML algorithms, namely logistic regression (LR), Naïve Bayes (NB), decision tree (DT), support vector machine (SVM), artificial neural network (ANN) and two ensemble learning methods of random forest (RF) and extreme gradient boosting (XGB) based on epidemiological survey data. Third, we used SHapley Additive exPlanations (SHAP) to examine the key predictors and how they influenced the progression of frailty from global and local perspectives to promote tailored intervention strategies.

## Methods

### Study participants

Data was collected from the Chinese Longitudinal Healthy Longevity and Happy Family Study (CLHLS-HF), a nationally representative social science survey, conducted by the Center for Healthy Aging And Development Studies, National School of Development of Peking University [16], and was approved by the Institutional Review Board, Duke University (Pro00062871), and the Biomedical Ethics Committee, Peking University (IRB00001052–13074). All participants provided written, informed consent. The baseline survey was conducted in 1998 and the follow-up surveys were conducted in 2000, 2002, 2005, 2008–2009, 2011–2012, 2014 and 2017–2018 in randomly selected about half of the counties and city districts in 23 Chinese provinces consisting of older adults aged 65 and above and their children aged 35–64 [17].

The latest 6 waves targeted people aged 65 years and above were selected for analysis, the first 2 waves (1998 and 2000) were excluded for only enrolling participants older than 80 years. Additionally, participants were restricted to be followed for at least 3 waves from the baseline of 2002 for analysis of frailty trajectories. Finally, 4083 subjects were included. The follow-up information of CLHLS-HF and the sample selection of this study are presented in Supplementary Fig. S1.

### Frailty assessment

We constructed FI following the standard procedure published by Searle et al. [18] The FI in the current study was derived from major domains of health (i.e., ADLs, IADLs, cognition, functional limitations, and self-reported health) consisting of 38 items associated with adverse health outcomes [19]. We used the sum of deficit points that appeared in the individual to divide the total number of deficits considered as an indicator of FI. If there were missing items (allowed within 1/3 of total items), then will be excluded from both the denominator and the numerator [20]. Ultimately, the FI scores were used to identify the potential trajectories of frailty in the current analysis. The description of items and coding instructions for the current study were listed in Supplementary Table S1.

### Trajectories of frailty

In the field of medical research, there are many time-dependent variables and developmental trajectory can describe the change of variables over time and dynamically reflect the characteristics of these variables. Typical traditional methods including hierarchical modeling and latent curve analysis were commonly used to analyze developmental trajectories based on the continuous distribution function [21, 22]. However, they usually obtained the average trajectory of the variables without the consideration of individual heterogeneity among the whole population. GBTM was known as a semi-parametric mixture model. The basic principle of GBTM is to fit model trajectories with polynomial functions of age or time and GBTM is mainly used to analyze longitudinal data to identify clusters of individuals with similar trajectories and subgroups with different trajectory types [23]. In the present study, we extracted the relevant indicators from questionnaires in different follow-up years to establish a repeated FI index, and age in follow-up years was defined as the timescale. According to prior study, we tried 1–6 trajectories with the polynomial model (up to cubic model) to identify the latent subgroups of frailty trajectories [24]. The optimal number of subgroups was determined by the results of model fitting including Bayesian information criteria (BIC), Akaike’s information criterion (AIC) and the proportion of the smallest class (with more than 5% of all the participants for generalization) [25]. AIC and BIC were used to measure the variation between predicted and true values by the fitting model and the lower value of AIC and BIC indicated better model fitting. Furthermore, individual was assigned to the class with the average posterior probability (APP) greater than 70% indicating the model had good fit. In the current study, GBTM was applied to identify the sub-group of frailty trajectories of the target population (*N* = 4083) with at least three waves of data from 2002 to 2018 in stage 1, describing variation within-person and groups. Given that the number of follow-up waves toward FI may affect the formation of the frailty trajectory, we further described the trajectory of frailty development for the study subjects with complete six waves data (*N* = 617) to validate the stability of the trajectory results.

### Measurement of predictors

Predictors of machine learning in this study covered sociodemographic characteristics, lifestyles, self-reported health, and objective examination. Previous study have confirmed that missForest outperforms other methods including *k*-nearest neighbor (KNN impute) and multiple imputation (MICE) in missing value imputation [26]. So in the current study, missForest was used to fill the missing values of variables. MissForest is a non-parametric missing value imputation method for mixed-type data involving continuous variables as well as categorical variables based on a random forest. The main principle of missForest is to address the missing data by training random forest on observed values in the first step, then give prediction of the missing values iteratively. More detailed description of variables was presented in Supplementary Methods S1, and the measurements of variables were listed in Supplementary Table S2.

### Feature selection

For pursuing model parsimony and excluding irrelevant variables related to health, feature selection was recognized as a good choice with avoiding over-fitting of prediction models [27]. We used recursive feature elimination (RFE) with 10-fold cross-validation based on random forest to select the most relevant features in the training dataset, ranking features by the measure of variable importance, iteratively eliminating the least important features, and re-fitting the model to search for optimal number of features remained in the model by repeating the process [28].

### Machine learning classifiers

In this study, 7 machine learning methods including naïve bayes (NB), logistic regression (LR), decision tree (DT), support vector machine (SVM), artificial neural network (ANN), random forest (RF), and extreme gradient boosting (XGB) were used to predict the frailty trajectories in stage 2. NB, a simple model based on the knowledge of probability theory and statistics. It is used to estimate the likelihood of things happening based on a priori knowledge [29]. LR, considered as a special case of a generalized simple linear model, can transform linear regression values into the range of (0,1) by the 𝑠𝑖𝑔𝑚𝑜𝑖𝑑 function, and further the continuous regression is transformed into a binary classification task by setting a threshold. LR was usually selected as a base model for comparison [30]. DT is highly popular in medical decision making, and it is similar to a tree structure and consists of a series of nodes and branches. The classification task is usually done by a series of rules starting from the root node. It can code with nonlinear relationships among multiple variables and showed strong interpretability by separating each branch of the tree into increasingly smaller leaves based on various features [31]. SVM, favored with solid statistical theory, maps data to a higher dimension to find an optimal decision hyperplane that maximizes the separation between two classes of data, and it can overcome the problem of linear inseparability [32]. ANN, comprised of input units, hidden units, and output units, is one of a mathematical model belonged to deep learning, it can simulate the response mechanism of the human brain nervous system to external stimulus. Neurons are the most basic units of data processing. First, data is entered into the input layer, the information is passed to each neuron in the hidden layer through the connection weights of each node. Next, the data is further weighted, summed and transformed by the activation function. Last, the processed information is further fed to the output layer and further processed to be specific output. ANN is able to detect complex relationships between dependent and independent variables [33]. Lessons from previous studies showed that the performance of single weak model can be improved by combination of multiple learners, which was called ensemble methods. RF is one of the ensemble methods, known as bagging, and it consists of a number of decision trees in parallel, and the predictions are mostly voted by numerous decision trees [34]. XGB is a strong classifier of decision-tree-based ensemble method by integrating multiple weak learners (boosting). It can prevent overfitting with complexity regularization [35].

### Derivation and evaluation of prediction models

**Fig. 1:**
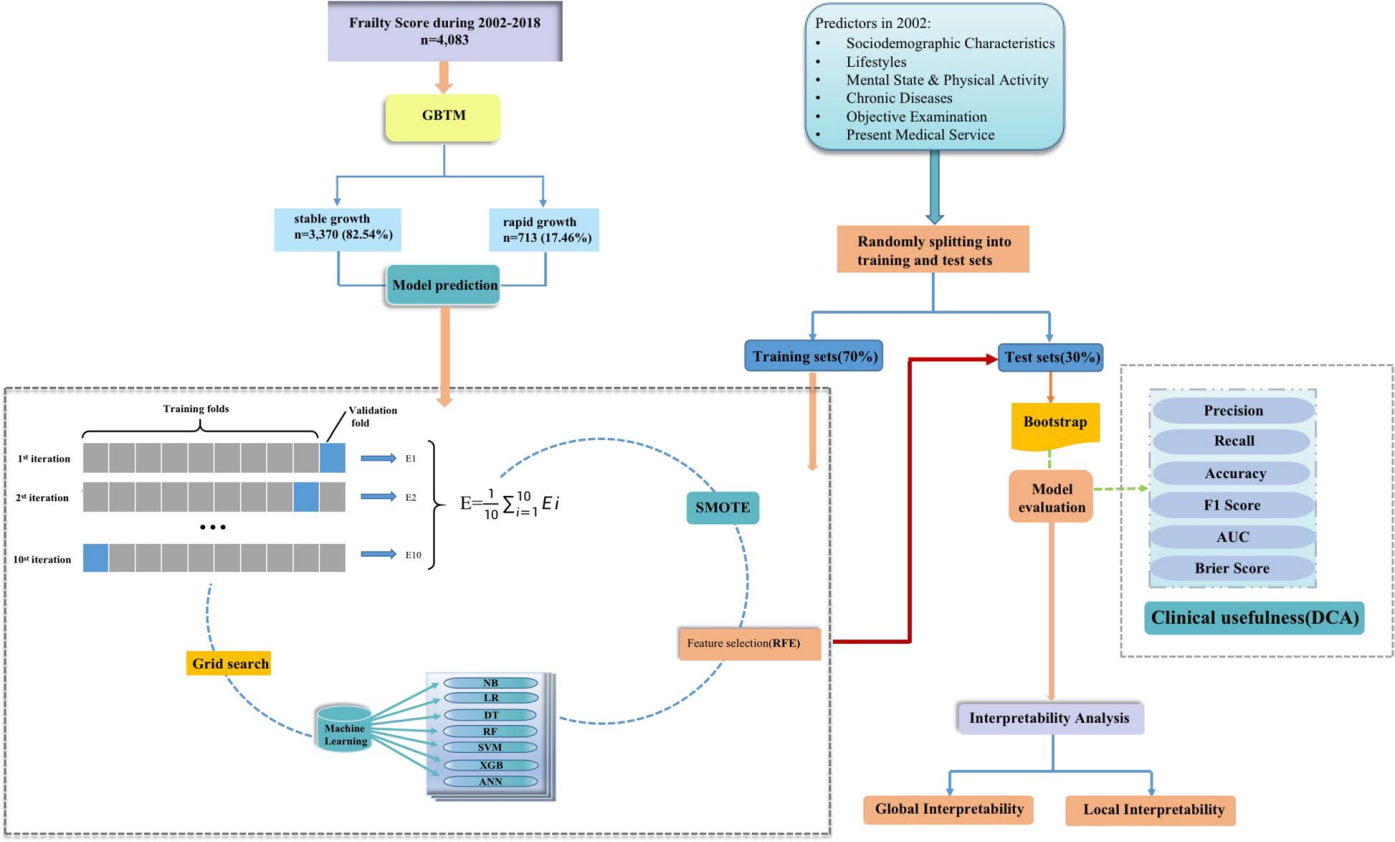
Flow chart of the study. NB: Naïve Bayes; LR: Logistic regression; DT: Decision tree; RF: Random forest; SVM: Support vector machine; XGB: Extreme Gradient Boosting; ANN: Artificial neural network

For model development, we applied grid search to achieve hyperparameter optimization and to prevent overfitting [36]. Firstly, the datasets were randomly split into training set (70%) and test set (30%). In the training set, we combined 10-fold cross validation and grid search to determine the best hyperparameters based on the best accuracy in validation set. Finally, the test set were subjected to 1000 times bootstrap resampling for internal validation [37]. The model performance can be lowered by class imbalance, therefore, synthetic minority over-sampling technique (SMOTE), combining oversampling with under-sampling, was applied for resampling by analyzing minority sample then adding new cases to dataset in the training set [38]. Multi-dimensional metrics including discrimination, calibration and clinical usefulness were considered [39]. In terms of discrimination, the evaluation metrics covered: (1) weighted precision, calculates precision for each label, and then finding their average weighted values (the number of true instances for each label), (2) weighted recall, calculates recall for each label, and then finding their average weighted values (the number of true instances for each label), (3) balanced accuracy, calculates the  average of recall obtained on each class, which is suitable for class imbalance, (4) weighted F1 score, F1 score is the synthesis of precision and recall, and weighted result was calculated based on F1 score of each label, (5) area under the receiver operating characteristic curve (AUROC), ranging from 0 to1, reflects the overall classification performance, the closer to 1, the more favored model performance. The brier score was used to examine both model discrimination and calibration [40], representing the differences between those predictions and their corresponding event scores. Obviously, the lower brier score, the better model accuracy. As for clinical usefulness, the decision curve analysis (DCA) was used to quantify the net benefit of model implementation in practice determined by the difference between the expected benefit and the expected harm with various threshold provided for clinical decision [41]. The whole work flow was shown in Fig. 1.

### Machine learning interpretation

More sophisticated algorithm usually accompanying opaque deduced outcomes like a black box [42]. So we used SHapley additive explanations (SHAP) to help clinicians and community workers better understand the model and promote the transition from model construction to practice. SHAP can tell the importance of various features from global aspect and peer into how every single feature influence the outcome [43]. We further used restricted cubic spline to explore the association between significant predictors and rapid-growth frailty trajectory.

### Statistical analysis

Continuous variables were presented by mean ± standard deviation for normal distribution or presented by median and inter-quartile range for skewed distribution. Categorical variables were given percentages. The differences of baseline characteristics among different trajectories were analyzed by *t* test or Wilcoxon test for continuous variables and chi square test or Fisher exact test for categorical variables. All the above analyses were performed by SPSS 25.0. Frailty trajectories were identified by SAS 9.4.0. The process of prediction was performed with Python 3.7.6. R (4.2.0) was applied to plot Restricted cubic spline (RCS). A 2-sided probability value of *p* < 0.05 was considered to be statistically significant.

## Result

### Heterogeneous trajectories of frailty

**Fig. 2:**
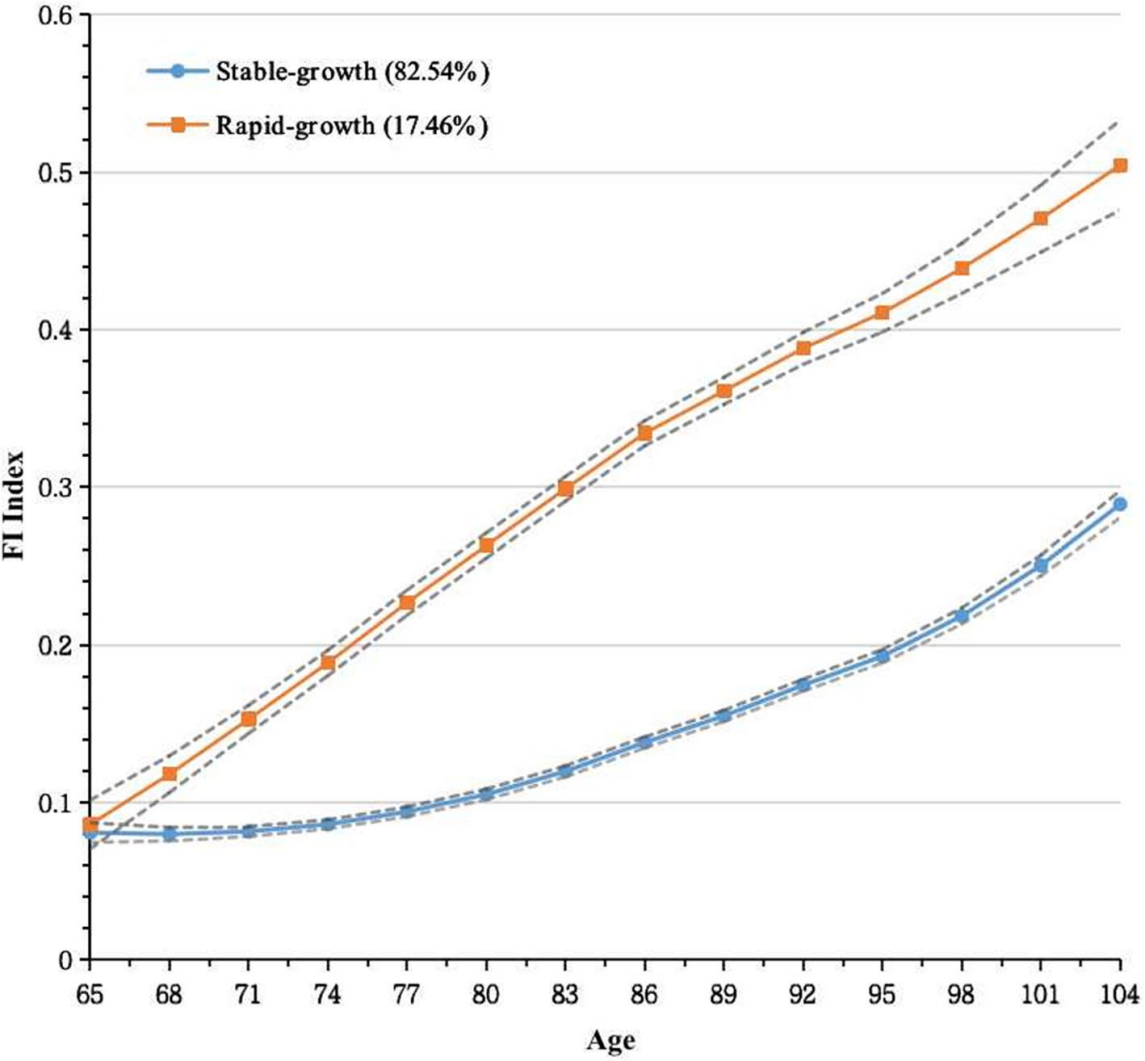
Heterogenous trajectories of older adults satisfied with inclusion criteria for at least three waves (*n* = 4083)

On inspection of Supplementary Table S3 and Table S4, 2-class with both quadratic form showed relative lower BIC (14,639.21), and AIC (14,664.47), the proportion of smallest class was over 5% and the posterior probability for every group was more than 70%. Although, 1-class showed relatively lower value of AIC and BIC, it was more significant to identify the heterogeneous frailty trajectories. Thus, two trajectories were identified for the development of frailty among older adults. Figure 2 shows two longitudinal patterns of frailty, plotted by current age at each visit, based on FI: class 1, “stable-growth” (*n* = 3370, 82.54%); class 2, “rapid-growth” (*n* = 713, 17.46%). The detailed parameters and maximum likelihood estimation for the final two-group trajectory model are summarized in Supplementary Table S4. The sensitivity analyses also identified similar frailty trajectories as shown in Supplementary Fig. S2.

Frailty index was generated based on the number of deficits present in an individual divided by the total number of deficits possible, ranging from 0 to 1. Two distinct trajectory classes were identified: “stable-growth”, “rapid-growth”. Stable-growth described that the value of FI increased stably, rapid-growth captured that the change of FI increased unsteadily for long-term trend with a relative steep speed. The solid lines represent the means, and the dashed lines represent the 95% confidence intervals of the mean.

### Baseline characteristics of study population

A total of 27 variables were selected by proposed RFE with ten-fold cross-validation (Supplementary Fig. S3). The average age was 77. 0 years of all selected subjects (*n* = 4083) and female accounted for 52.0%. 20.0% of them lived in city, 46.2% never received formal education. 50.9% were in married and most of them were living with chronic disease. The baseline average MMSE, ADL and IADL scores were 27.22, 11.80 and 14.02 respectively. Supplementary Table S5 shows the results of comparisons of baseline characteristics between analytical sample and drop-out sample among those aged 65 years or older in 2002. The comparisons of baseline characteristics between the two trajectory classes are presented in Supplementary Table S6. The enrolled participants were more inclined to settle in rural, have relative lower level of education, have more alcohol consumption and a higher rate of living with arthritis and hypertension. In addition, they had a slightly higher economic status and much more frequent of entertainment activities. They performed better in cognition ability and physical function.

### Performance of prediction models

**Fig. 3:**
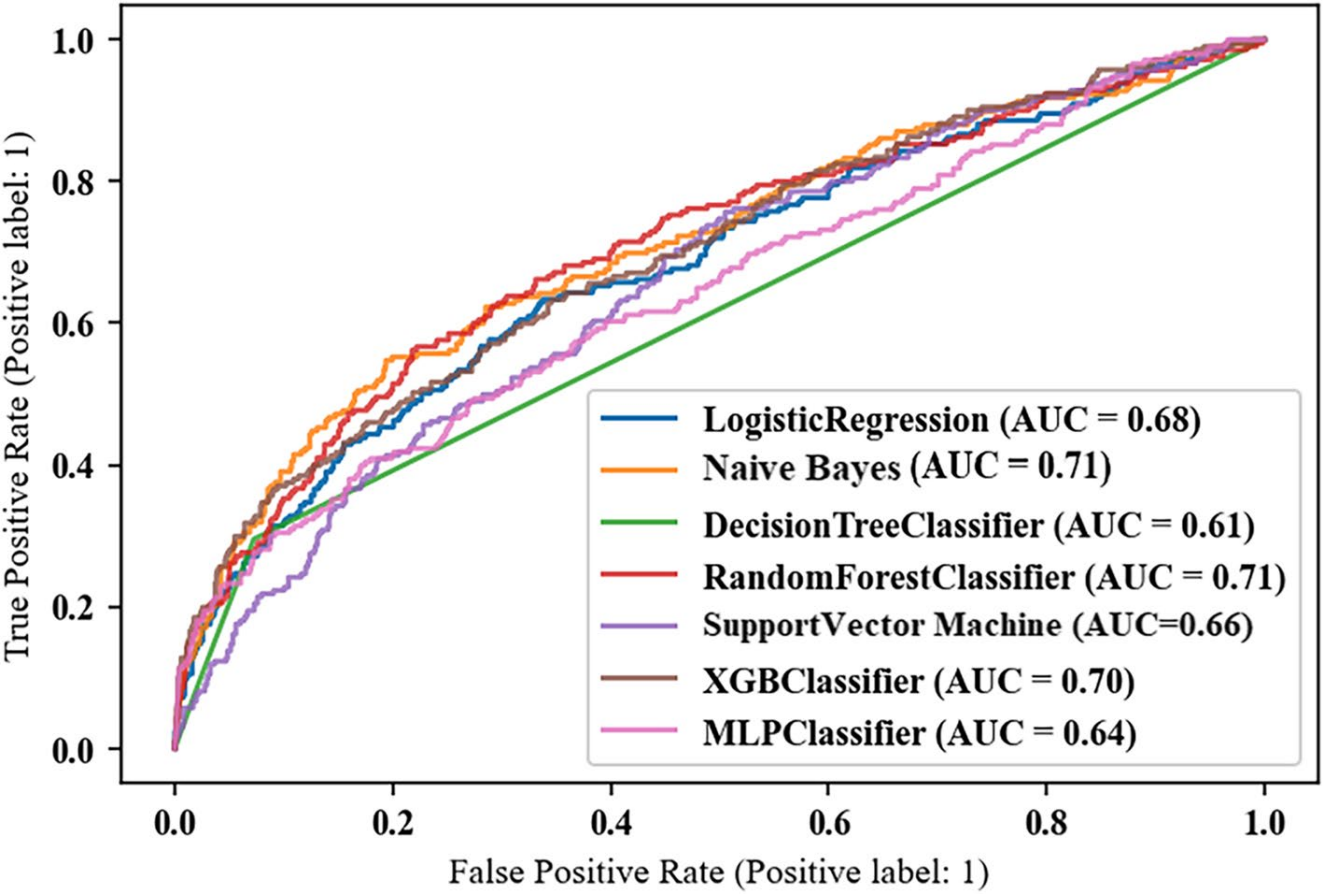
Area under the receiver operating characteristic curves (AUCs) of models

**Fig. 4:**
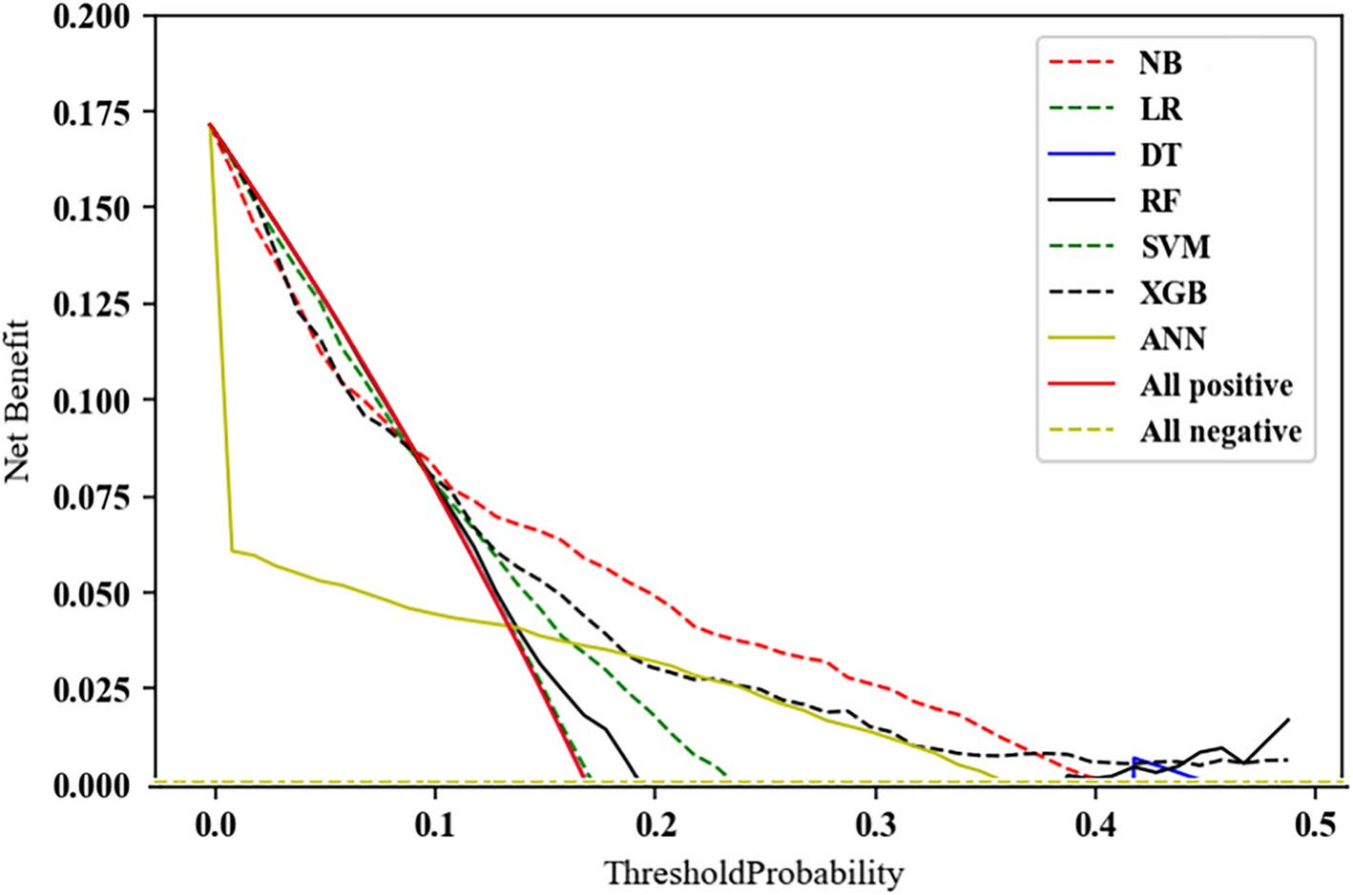
Decision curve of prediction models and net benefit of each model. NB: Naïve Bayes; LR: logistic regression; DT: decision tree; RF: random forest; SVM: support vector machine; XGB: extreme gradient boosting; ANN: artificial neural network; All positive: the net benefit of providing screen for all subjects (all subjects would be positive); All negative: the net benefit of providing screen for all subjects (all subjects would be negative)

Table 1 presents the weighted precision, weighted recall, balanced accuracy, weighted F1 score, AUROC and brier score of ML algorithms in test set. The accuracy of all models ranges from 0.60 ~ 0.66. RF reached the best recall of 0.845, the best precision of 0.820 and the best F1 score of 0.816, respectively. Additionally, RF achieved 0.702 in AUROC and relative lower brier score of 0.143. Area under the receiver operating characteristic curves (AUCs) of all prediction models were presented in Fig. 3. Figure 4 presented the results of decision curve analysis for all models. Specifically, the net benefit of decision curve is the sum of the gain value of the intervention for the corresponding true-positive population at each threshold and the loss value of the intervention for the false-positive population. All positive and all negative line represents the net benefit of providing screen for all subjects, assuming that all subjects would be positive and negative respectively. The corresponding net benefits are varying under different thresholds. Our results showed that if the threshold probability is higher than 10%, a certain intervention for the population identified as at risk under that threshold has more benefit than an intervention for all subjects or no subjects at all. Generally, NB was superior to others in a wide range of thresholds.

**Table 1:** Performance of machine learning algorithms for frailty trajectories prediction

	Accuracy	Recall	Precision	F1 Score	AUROC	Brier Score
NB	0.645 (0.610, 0.680)	0.810 (0.787, 0.832)	0.802 (0.777, 0.827)	0.805 (0.782, 0.829)	0.722 (0.680, 0.764)	0.161 (0.143, 0.179)
LR	0.664 (0.627, 0.702)	0.701 (0.674, 0.728)	0.798 (0.773, 0.823)	0.733 (0.709, 0.757)	0.728 (0.687, 0.769)	0.205 (0.196, 0.213)
DT	0.612 (0.580, 0.643)	0.819 (0.798, 0.839)	0.794 (0.769, 0.820)	0.802 (0.779, 0.826)	0.612 (0.580, 0.643)	0.208 (0.202, 0.214)
RF	0.610 (0.580, 0.639)	0.845 (0.825, 0.864)	0.820 (0.794, 0.846)	0.816 (0.791, 0.841)	0.702 (0.659, 0.745)	0.143 (0.134, 0.151)
SVM	0.596 (0.560, 0.632)	0.723 (0.697, 0.748)	0.765 (0.737, 0.793)	0.740 (0.716, 0.765)	0.661 (0.619, 0.703)	0.181 (0.167, 0.195)
XGB	0.599 (0.580, 0.639)	0.833 (0.825, 0.864)	0.802 (0.794, 0.846)	0.806 (0.791, 0.841)	0.677 (0.659, 0.745)	0.134 (0.134, 0.151)
ANN	0.595 (0.563, 0.628)	0.796 (0.773, 0.819)	0.777 (0.750, 0.804)	0.785 (0.760, 0.810)	0.640 (0.595, 0.685)	0.193 (0.171, 0.215)

Note: *NB* Naïve Bayes, *LR* Logistic regression, *DT* Decision tree, *RF* Random forest, *SVM* Support vector machine, *XGB* Extreme Gradient Boosting, *ANN* Artificial neural network, *Accuracy* refers to balanced accuracy, *Precision*, *Recall, and F1 score* refer to the weighted results.

### Interpretability analysis of prediction models

**Fig. 5:**
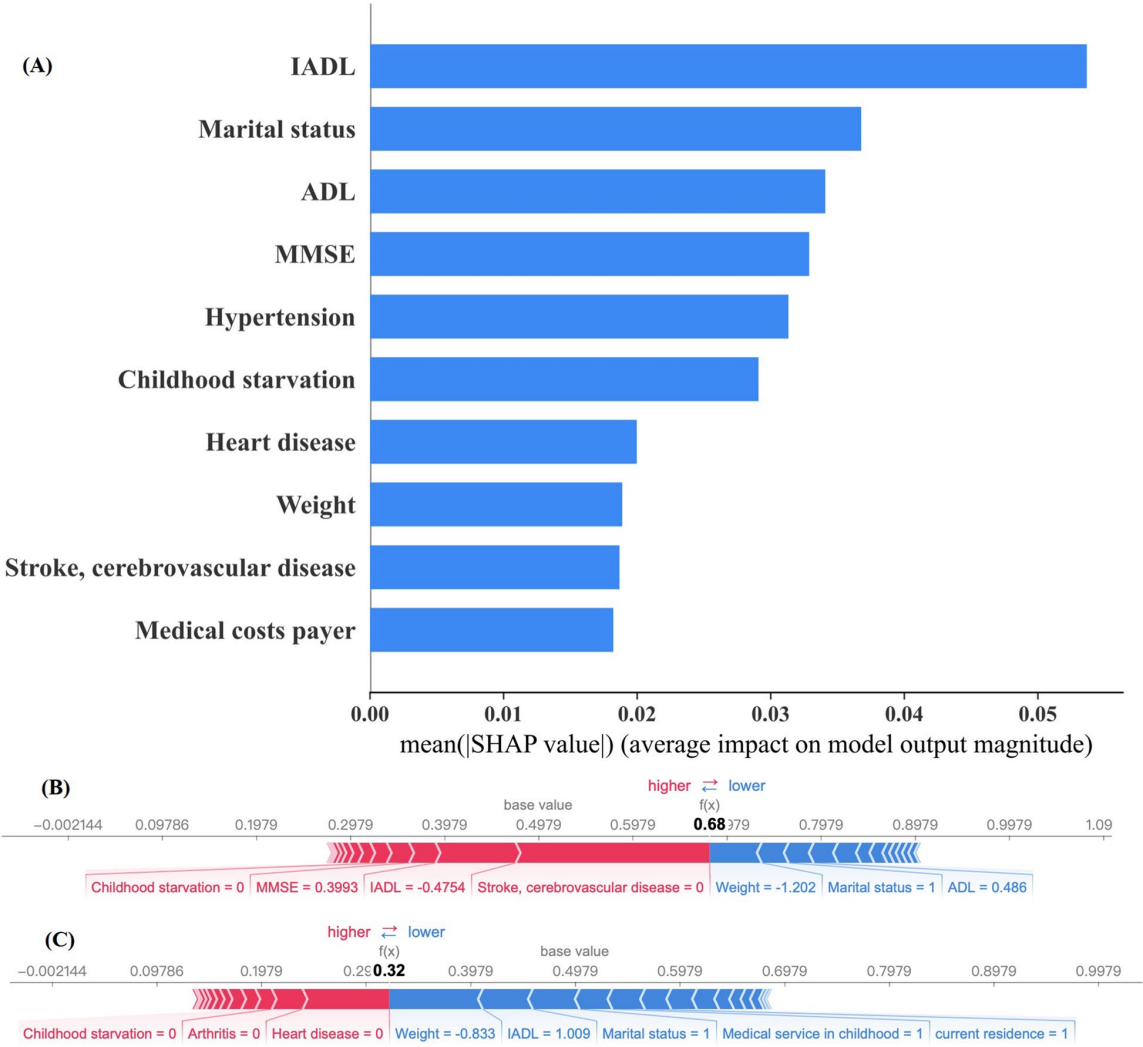
Interpretability analysis with SHapley Additive exPlanations. **A** Each row represents a feature, the x-axis represents the SHAP value. **B** Single prediction for sample randomly selected (true positive). **C** Single prediction for sample randomly selected (true negative)

**Fig. 6:**
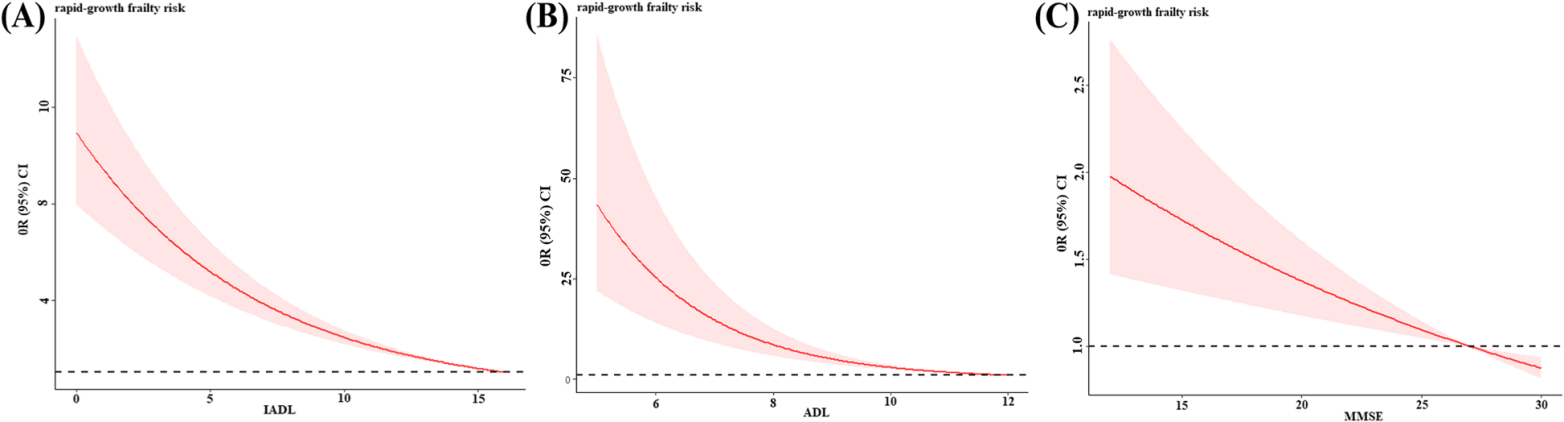
Restricted cubic spline (RCS), based on logistic regression to analyze the relationship between important variables including IADL, ADL and MMSE and frailty trajectory

Overall, RF model performed excellent in current study. So, we provided explanations for RF model by SHAP to help to learn how single variable contributed to a certain outcome and to examine crucial factors. SHAP was used to translate the predicted value of the model as the sum of the attribution values (shap values) of each input feature. According to summary plot (Fig. 5A), we can see the top 10 features with larger mean absolute of shape value from global measure, especially for physical function (IADL and ADL), marital status, cognition function and living with hypertension, they greatly determined the output of the model, which can be used as a reference for prevention. Further, we gave single-prediction explanations by SHAP force plot to visualize the transition from base value to output value driven by single feature allowing for personalized decision-making. From the individual-level interpretability analysis of SHAP, we can see that each feature has its own attributed value, and that all attributed value drove the model’s predictions from the base value to the final model output. The horizontal axis position indicates the impact of each feature on output value. Features that increase the predicted value from base value were denoted by red block, while features that pushed the predicted value down from base value were denoted by blue block. Figure 5B and C showed single prediction for both truly positive and truly negative samples randomly selected from test set. Further, based on the restricted cube plot (Fig. 6), it is known that whatever decline in physical function or cognition function will increased the risk of rapid-growth trajectory of frailty, especially the decline in physical function was more sensitive to the frailty trajectory than cognitive function decline in line with global SHAP analysis. Being similar to the global interpretability analysis of SHAP, the logistic regression (listed in Tables S7) results also implied that living with stroke/cerebrovascular disease, hypertension, heart disease and physical function decline may increase the risk of frailty.

## Discussion

According to the results of GBTM, two frailty trajectories were identified and majority of the older adults will keep a relative stable growth of frailty trend in over 16 years. Then we used machine learning to make prediction of frailty trajectory with easily available predictors compared to blood biomarkers and imaging data. Our proposed machine learning models achieved high performance in distinguishing the two types of frailty trajectories with the AUCs closing to 0.7, indicating that ML could serve as a feasible screening tool, and our models were promising to be employed to give frailty trajectory prediction based on simple epidemiological data so as to identify older adults at high-risk and significant modifiable factors such as diet, daily activities and economic situation etc., which may facilitate early effective intervention to delay or prevent the occurrence or progression of adverse event.

It is worthwhile to conduct further discussion on the inconsistency of model performance and clinical decision curves. Our results showed that RF performs relatively better in terms of discrimination and calibration metrics, but its clinical decision benefit was not the highest. In view of previous studies, discrimination and calibration analyses are the evaluation of the model accuracy independent to its clinical value [44]. Thus, we should be more cautious in making clinical decisions of prediction models. The SHAP summary plot showed that physical and cognition function, marital status, living with hypertension, heart disease, stroke/cerebrovascular disease, suffering from childhood starvation, weight, and medical cost payer were top 10 important variables. In clinical decision-making, clinicians should prioritize treatment for physical function and cognitive function recovery and give essential consideration of co-morbid chronic diseases. According to the analysis results, it is not difficult to find that the SHAP value and the odds ratios based on binary logistic regression identified several shared significant factors such as physical function including IADL and ADL, marital status, heart disease, hypertension, and stroke. However, some crucial factors such as MMSE appeared in SHAP analysis but its corresponding odds ratios were absence of statistical significance. The disparity may be derived by the prevalence of variables. The odds ratios are related to the distribution of variables in the population, whereas the SHAP values are the average impact across the whole population [45]. According to SHAP summary plot, IADL, ADL and MMSE are all the strong factors related to frailty consistent with the criteria of FI. Given the parameters including in FI were ongoing the absence of authoritative convention [46], our results put forward a proposal that FI can be calculated by assigning deficit accumulation distinct weights reference to feature importance. Thereby, physical function and cognition function cannot be ignored, timely screening should be implemented. Further, aging accelerated the progression of frailty despite the status of physical function. In turn, early intervention on frailty can help improve physical function and delay or prevent the onset of dependency.

There were some modifiable risk factors according to our findings such as less social activity, unhealthy lifestyle, poor dietary habit and adverse childhood experiences which can be regard as prevention target to lower the risk of frailty worsening [46, 47]. It should be encouraged to carry out various social activities for older adults with more young people involved in community or nursing home, health education and health screening should also be implemented further. Additionally, older adults living with multimorbidity were more vulnerable. However, nearly half of the patients showed a negative attitude towards the treatment of chronic diseases, on the other hand, few clinical decisions focus on the condition of multimorbidity, which suggested us that clinicians should evaluate the patient’s health comprehensively when making decision and establish good communication with patients to improve their compliance [48]. We also call for timely screening for cognitive decline, physical limitations and frailty in community and in clinic settings offering suggestions in therapy.

All in all, the current study verified the potential of future projections that combining machine learning and available health data as no time-consuming screening tools to promote frailty assessment, provided reference to individualized, flexible prevention and treatment considering individual heterogeneity. A bunch of studies tried to achieve automatic diagnosis with ML algorithms, however lack of adequate explainability made it a crucial barrier toward clinical use [49]. Our study incorporated ML model with SHAP making the prediction more transparent and reliable by giving explanation of risk variables and displaying individual prediction. Nevertheless, more modifiable factors related to frailty needed to be investigated, and further shed in light whether early prevention could alter the trend of frailty, more importantly, how to translate from the research of secular trends in frailty to practice is a crucial challenge in future.

This study has some limitations that warrant discussion. Firstly, the data was drawn from a retrospective national survey, implying loss to follow-up is inevitable, so we applied missForest imputation approach to deal with the incomplete data. Although missForest performed better than mean value of variables, it cannot eliminate the deviation rooting in data quality completely. Secondly, the sample size was relatively small, to solve this problem, we combined ten folds cross validation in training set and bootstrap resampling in test set to avoid overfitting and make full use of sample information. Thirdly, GBTM model was used to fit the frailty trajectory of the elderly population trying from groups of 1–6, and the final trajectory-group was determined by combining the model fitting results (AIC and BIC) and other evaluation metrics. According to our fitting results, single class showed the lowest values of AIC and BIC, but previous studies have examined the heterogeneity of frailty trajectories and distinguishing different categories of frailty trajectories makes more clinical significance for intervention treatment. Finally, 2-group of frailty trajectory was selected. Fourth, the absence of more complicated information such as biological data may limit the model performance even wider model application. Moreover, further study should focus on external validation in a more general population to break down the barriers to the transformation from research to clinical practice.

## Conclusion

This study described the progression of frailty and gave possible frailty prediction based on explainable machine learning. The results demonstrated the potential of machine-learning algorithms in identifying higher-risk subgroup of frailty trajectories, which could benefit to optimize health resource utilization. Interpretability analysis could help health care provider to better understand and trust the possible pathway of precision prevention, treatment, and management of older adults in need.

## Supplementary Information

**Additional file 1: Supplementary Methods S1.** Detailed description of variables. **Supplementary Table S1.** Deficits of frailty index and corresponding coding based on the Chinese Longitudinal Healthy Longevity and Happy Family Survey. **Supplementary Table S2.** Measurement of variables in 2002. **Supplementary Table S3.** Performance of trajectories fitting models. **Supplementary Table S4.** The parameters of Frailty trajectory. **Supplementary Table S5.** Characteristics comparison between analytical sample and drop-out sample among those aged 65 years or older in 2002. **Supplementary Table S6.** Selected characteristics comparison between trajectories classes in 2002. **Supplementary Table S7.** Comparison of sample characteristics by multivariate logistic model between stable-growth and rapid-growth frailty trajectories. **Supplementary Fig. S1.** Follow-up information of CLHLS-HF and the sample selection. **Supplementary Fig. S2.** Heterogenous frailty trajectories classes for at least six waves. **Supplementary Fig. S3.** Feature selection based on recursive feature elimination.

## Data Availability

The CLHLS-HF data is freely available at https://opendata.pku.edu.cn/.

## Abbreviations

FI: Frailty Index
GBTM: Group-Based Trajectory Modeling
ML: Machine Learning
LR: Logistic Regression
NB: Naïve Bayes
DT: Decision Tree
SVM: Support Vector Machine
ANN: Artificial Neural Network
RF: Random Forest
XGB: Extreme Gradient Boosting
SHAP: SHapley Additive exPlanations
AUROC: Area Under the Receiver Operating Characteristic Curve

## References

[1] Morley JE, Vellas B, Abellan van Kan G, Anker SD, Bauer JM, Bernabei R, Cesari M, Chumlea WC, Doehner W, Evans J (2013). Frailty consensus: a call to action. J Am Med Dir Assoc.

[2] Collard RM, Boter H, Schoevers RA, Oude Voshaar RC (2012). Prevalence of frailty in community-dwelling older persons: a systematic review. J Am Geriatr Soc.

[3] Pereira AA, Borim FSA, Aprahamian I, Neri AL (2019). Comparison of two models of frailty for the prediction of mortality in Brazilian community-dwelling older adults: the FIBRA study. J Nutr Health Aging.

[4] Fried LP, Tangen CM, Walston J, Newman AB, Hirsch C, Gottdiener J, Seeman T, Tracy R, Kop WJ, Burke G (2001). Frailty in older adults: evidence for a phenotype. J Gerontol A Biol Sci Med Sci.

[5] O'Caoimh R, Sezgin D, O'Donovan MR, Molloy DW, Clegg A, Rockwood K, Liew A (2021). Prevalence of frailty in 62 countries across the world: a systematic review and meta-analysis of population-level studies. Age Ageing.

[6] Dent E, Martin FC, Bergman H, Woo J, Romero-Ortuno R, Walston JD (2019). Management of frailty: opportunities, challenges, and future directions. Lancet.

[7] Beard JR, Officer A, de Carvalho IA, Sadana R, Pot AM, Michel JP, Lloyd-Sherlock P, Epping-Jordan JE, Peeters G, Mahanani WR (2016). The world report on ageing and health: a policy framework for healthy ageing. Lancet.

[8] Verghese J, Ayers E, Sathyan S, Lipton RB, Milman S, Barzilai N, et al. Trajectories of frailty in aging: prospective cohort study. PLoS One. 2021;16(7). 10.1371/journal.pone.0253976 PMC8274857 34252094

[9] Sinclair A, Morley J (2013). Frailty and diabetes. Lancet.

[10] Kojima G, Avgerinou C, Iliffe S, Walters K (2018). Adherence to Mediterranean diet reduces incident frailty risk: systematic review and Meta-analysis. J Am Geriatr Soc.

[11] Feng Z, Lugtenberg M, Franse C, Fang X, Hu S, Jin C, Raat H (2017). Risk factors and protective factors associated with incident or increase of frailty among community-dwelling older adults: a systematic review of longitudinal studies. PLoS One.

[12] Tarekegn A, Ricceri F, Costa G, Ferracin E, Giacobini M. Predictive modeling for frailty conditions in elderly people: machine learning approaches. JMIR Med Inform. 2020;8(6). 10.2196/16678 PMC7303829 32442149

[13] Deo RC (2015). Machine learning in medicine. Circulation.

[14] Schwalbe N, Wahl B (2020). Artificial intelligence and the future of global health. Lancet.

[15] Vellido A (2020). The importance of interpretability and visualization in machine learning for applications in medicine and health care. Neural Comput Applic.

[16] Yi Z: Introduction to the Chinese Longitudinal Healthy Longevity Survey (CLHLS). In: *Healthy Longevity in China: Demographic, Socioeconomic, and Psychological Dimensions.* vol. 20; 2008: pp 23–38.

[17] Yi Z, Jr D, Vlosky DA, Gu D: Healthy longevity in China: demographic, socioeconomic, and Psychological Dimensions, vol. 20; 2008.

[18] Searle SD, Mitnitski A, Gahbauer EA, Gill TM, Rockwood K (2008). A standard procedure for creating a frailty index. BMC Geriatr.

[19] Gu D, Dupre ME, Sautter J, Zhu H, Liu Y, Yi Z (2009). Frailty and mortality among Chinese at advanced ages. J Gerontol B Psychol Sci Soc Sci.

[20] Stuck AK, Mangold JM, Wittwer R, Limacher A, Bischoff-Ferrari HA. Ability of 3 frailty measures to predict short-term outcomes in older patients admitted for post-acute inpatient rehabilitation. J Am Med Dir Assoc. 2021. 10.1016/j.jamda.2021.09.029 34687605

[21] Cohen-Addad V, Kanade V, Mallmann-Trenn F, Mathieu C, Assoc Comp M: Hierarchical Clustering: Objective Functions and Algorithms. In: *SODA'18: PROCEEDINGS OF THE TWENTY-NINTH ANNUAL ACM-SIAM SYMPOSIUM ON DISCRETE ALGORITHMS*. 2018: 378–397.

[22] Stamm KE, Harlow LL, Walls TA: An introduction to latent variable growth curve modeling: concepts, issues, and applications (2nd ed.). Struct Equ Model Multidiscip J 2007, 14:701–706.

[23] Nagin DS, Odgers CL: Group-based trajectory modeling in clinical research. (1548–5951 (Electronic)). 10.1146/annurev.clinpsy.121208.131413 20192788

[24] Welstead M, Luciano M, Russ TC, Muniz-Terrera G: Heterogeneity of Frailty Trajectories and Associated Factors in the Lothian Birth Cohort 1936. (1423–0003 (Electronic)). 10.1159/000519240 PMC9501780 34587617

[25] Muthén B, Muthén LK (2000). Integrating person-centered and variable-centered analyses: growth mixture modeling with latent trajectory classes. Alcohol Clin Exp Res.

[26] Stekhoven DJ, Bühlmann P: MissForest--non-parametric missing value imputation for mixed-type data. (1367–4811 (Electronic)). 10.1093/bioinformatics/btr597 22039212

[27] Wiemken TL, Kelley RR (2020). Machine learning in epidemiology and health outcomes research. Annu Rev Public Health.

[28] Kuhn M, Johnson K (2013). Applied predictive modeling.

[29] Tsangaratos P, Ilia I (2016). Comparison of a logistic regression and naive Bayes classifier in landslide susceptibility assessments: the influence of models complexity and training dataset size. Catena.

[30] Christodoulou E, Ma J, Collins GS, Steyerberg EW, Verbakel JY, Van Calster B (2019). A systematic review shows no performance benefit of machine learning over logistic regression for clinical prediction models. J Clin Epidemiol.

[31] Doupe P, Faghmous J, Basu S (2019). Machine learning for health services researchers. Value Health.

[32] Huang J-C, Tsai Y-C, Wu P-Y, Lien Y-H, Chien C-Y, Kuo C-F, Hung J-F, Chen S-C, Kuo C-H (2020). Predictive modeling of blood pressure during hemodialysis: a comparison of linear model, random forest, support vector regression, XGBoost, LASSO regression and ensemble method. Comput Methods Prog Biomed.

[33] Kriegeskorte N, Golan T (2019). Neural network models and deep learning. Curr Biol.

[34] Breiman L (2001). Random forests. Mach Learn.

[35] Torlay L, Perrone-Bertolotti M, Thomas E, Baciu M (2017). Machine learning-XGBoost analysis of language networks to classify patients with epilepsy. Brain Inform.

[36] Ranjan GSK, Verma AK, Radhika S: K-Nearest Neighbors and Grid Search CV Based Real Time Fault Monitoring System for Industries. In: *2019 IEEE 5th International Conference for Convergence in Technology (I2CT): 29–31 March 2019 2019*. 1–5.

[37] Feng LH, Su T, Bu KP, Ren S, Yang Z, Deng CE, Li BX, Wei WY (2020). A clinical prediction nomogram to assess risk of colorectal cancer among patients with type 2 diabetes. Sci Rep.

[38] Hao M, Wang Y, SHJACA B (2014). An efficient algorithm coupled with synthetic minority over-sampling technique to classify imbalanced PubChem. BioAssay data. Anal Chim Acta.

[39] Huang CX, Li SX, Caraballo C, Masoudi FA, Rumsfeld JS, Spertus JA, Normand SLT, Mortazavi BJ, Krumholz HM (2021). Performance metrics for the comparative analysis of clinical risk prediction models employing machine learning. Circ Cardiovasc Qual Outcomes.

[40] Lian X, Zou J, Guo Q, Chen S, Lu L, Wang R, Zhou M, Fu Q, Ye Y, Bao C (2020). Mortality risk prediction in Amyopathic Dermatomyositis associated with interstitial lung disease: the FLAIR model. Chest.

[41] Zachariasse JM, Nieboer D, Oostenbrink R, Moll HA, Steyerberg EW (2018). Multiple performance measures are needed to evaluate triage systems in the emergency department. J Clin Epidemiol.

[42] The Lancet respiratory M: opening the black box of machine learning. Lancet Respir Med. 2018;6(11):801. 10.1016/S2213-2600(18)30425-9 30343029

[43] Wang K, Tian J, Zheng C, Yang H, Ren J, Liu Y, Han Q, Zhang Y (2021). Interpretable prediction of 3-year all-cause mortality in patients with heart failure caused by coronary heart disease based on machine learning and SHAP. Comput Biol Med.

[44] Vickers AJ: Decision analysis for the evaluation of diagnostic tests, prediction models and molecular markers. (0003–1305 (Print)). 10.1198/000313008X370302 PMC2614687 19132141

[45] van den Bosch T, Warps AK, tot Babberich MPM, Stamm C, Geerts BF, Vermeulen L, Wouters M, JWT D, Tollenaar R, Tanis PJ (2021). Predictors of 30-day mortality among Dutch patients undergoing colorectal Cancer surgery, 2011-2016. JAMA Netw Open.

[46] Kurkcu M, Meijer RI, Lonterman S, Muller M (2018). de van der Schueren MAE: the association between nutritional status and frailty characteristics among geriatric outpatients. Clin Nutr ESPEN.

[47] van der Linden BWA, Sieber S, Cheval B, Orsholits D, Guessous I, Gabriel R, von Arx M, Kelly-Irving M, Aartsen M, Blane D (2020). Life-course circumstances and frailty in old age within different European welfare regimes: a longitudinal study with SHARE. J Gerontol Series B Psychol Sci Soc Sci.

[48] Pagès-Puigdemont N, Mangues MA, Masip M, Gabriele G, Fernández-Maldonado L, Blancafort S, Tuneu L (2016). Patients’ perspective of medication adherence in chronic conditions: a qualitative study. Adv Ther.

[49] Deshmukh F, Merchant SS (2020). Explainable machine learning model for predicting GI bleed mortality in the intensive care unit. Am J Gastroenterol.

