# Supplementary material for: Latent trajectories of frailty and risk prediction models among geriatric community dwellers: an interpretable machine learning perspective
Source: BMC Geriatr. 2022 Nov 24;22:900. doi: 10.1186/s12877-022-03576-5 (PMC9700973; doi:10.1186/s12877-022-03576-5)
Supplement: Supplementary file 1 — Additional file 1: Supplementary Methods S1. Detailed description of variables. Supplementary Table S1. Deficits of frailty index and corresponding coding based on the Chinese Longitudinal Healthy Longevity and Happy Family Survey. Supplementary Table S2. Measurement of variables in 2002. Supplementary Table S3. Performance of trajectories fitting models. Supplementary Table S4. The parameters of Frailty trajectory. Supplementary Table S5. Characteristics comparison between analytical sample and drop-out sample among those aged 65 years or older in 2002. Supplementary Table S6. Selected characteristics comparison between trajectories classes in 2002. Supplementary Table S7. Comparison of sample characteristics by multivariate logistic model between stable-growth and rapid-growth frailty trajectories. Supplementary Fig. S1. Follow-up information of CLHLS-HF and the sample selection. Supplementary Fig. S2. Heterogenous frailty trajectories classes for at least six waves. Supplementary Fig. S3. Feature selection based on recursive feature elimination. [file 12877_2022_3576_MOESM1_ESM.docx]

## Supplementary Materials

**Latent trajectories of frailty and risk prediction models among geriatric community dwellers: an interpretable machine learning perspective**

**Supplementary Methods S1** Detailed description of variables

**Supplementary Table S1** Deficits of frailty index and corresponding coding based on the Chinese Longitudinal Healthy Longevity and Happy Family Study

**Supplementary Table S2** Measurement of variables in 2002

**Supplementary Table S3** Performance of trajectories fitting models

**Supplementary Table S4** The parameters of Frailty trajectory

**Supplementary Table S5** Characteristics comparison between analytical sample and drop-out sample among those aged 65 years or older in 2002

**Supplementary Table S6** Selected characteristics comparison between trajectories classes in 2002

**Supplementary Table S7** Comparison of sample characteristics by multivariate logistic model between stable-growth and rapid-growth frailty trajectories

**Supplementary Figure S1** Follow-up information of CLHLS-HF and the sample selection

**Supplementary Figure S2** Heterogenous frailty trajectories classes for at least six waves

**Supplementary Figure S3** Feature selection based on recursive feature elimination

**Supplementary Methods S1 Detailed description of variables**

For sociodemographic characteristics, age, sex (male, female), ethnicity (Han ancestry, minority), education (illiterate, literate), marital status (unmarried or separated or divorced or widowed, married), residence (city, town, rural), co-residence (yes, no), primary occupation (labor work, mental work),[1] retired (yes, no), continue working (yes, no), economic status (high level, average level, low level), medical service in childhood (inadequate, adequate), and be starving in childhood (yes, no) were included. For lifestyles, regular fruits intake (yes, no), regular vegetables intake (yes, no), current smoker (yes, no), ex-smoker (yes, no), current drinker (yes, no), ex-drinker (yes, no), current exerciser (yes, no), ex-exerciser (yes, no), regular physical labor (yes, no), leisure activity (including housework, personal outdoor activities, garden work, reading newspapers or books, raising domestic animals or pets, playing cards or mahjong, watching TV or listening to the radio, and taking part in some organized and religious activities) were considered. The responses of leisure activity were “almost every day (coded as 4)”, “not daily, but at least once a week (coded as 3)”, “not weekly, but at least once a month (coded as 2)”, “not monthly, but sometimes (coded as 1)”, and “never (coded as 0).” Then the frequency of leisure activities was divided into low frequency and high frequency according to the median of total score coded as 0 and 1.[2] Self-reported health was comprised of the question itself, physical and mental health. Physical health was evaluated by activity of daily living (ADL), the instrumental activity of daily living (IADL) and chronic diseases (yes or no) including hypertension, diabetes, heart disease, stroke/cerebrovascular disease, respiratory diseases, pulmonary tuberculosis, cataract, glaucoma, cancer, gastric or duodenal ulcer, Parkinson's disease, bedsore, arthritis, and dementia. ADL ability was measured through the Katz Index of Activities of Daily Living scale (Cronbach's a = 0.87).[3, 4] IADL was considered more complex than ADL developed by Lawton and Brody.[5] ADL was consisted of the 6 daily activities (bathing, dressing, continence, using the toilet, indoor transferring, and feeding themselves), and IADL was measured by the 8 instrumental activities (shopping, cooking, visiting neighbors, doing laundry, walking continuously for 1 km, continuously crouching and standing up 3 times, lifting a weight of 5 kg, and taking public transportation). Each item was coded as 2 (“independently”), 1 (“with part assistance”), or 0 (“with complete assistance”). So higher ADL score (0-12) and IADL score (0-16) indicated better physical condition. The mental health was described by psychological well-being (PWB) and cognition status in CLHLS-HF. PWB was measured by 7 items containing four positively-oriented questions and three negatively-oriented questions. Specifically, if the item is helpful to PWB, we coded as follows: 5 (“always”), 4 (“often”), 3 (“sometimes”), 2 (“seldom”), and 1 (“never”); and if the item is harmful to PWB, we coded in an opposite way. The total score ranges from 7 to 35, and the higher score for PWB the better well-being.[6, 7] The Chinese version of the Mini-Mental State Examination (MMSE), evolving from the questionnaire developed by Folstein and colleagues with the consideration of Chinese culture and socioeconomic characteristics,[8] was used to assess the cognition of participation and has been proven to have satisfied reliability and validity for older Chinese adults.[9] MMSE involving 7 aspects of orientation, food counting within one minute, memory, calculation, drawing, recall and language, as a total of 24 items. Participants were expected to perform better with higher score ranging from 0 to 30. Missing values of variables were filled by the missForest approach, categorical variables were processed by one-hot encoding, and numeric variables were mapped to 0-1 to help model convergence and improve model accuracy.[10]

**Table S1** Deficits of frailty index and corresponding coding based on the Chinese Longitudinal Healthy Longevity and Happy Family Study

| Description | Assigned values (value of 1 indicates a deficit and 0 indicates no deficit) | | | |
| --- | --- | --- | --- | --- |
| Visit neighbors by oneself | Independently=0 | Need some help=0.5 | Unable=1 |  |
| Shop by oneself if necessary | Independently=0 | Need some help=0.5 | Unable=1 |  |
| Cook meals by oneself if necessary | Independently=0 | Need some help=0.5 | Unable=1 |  |
| Wash clothing by oneself | Independently=0 | Need some help=0.5 | Unable=1 |  |
| Walk continuously for 1 kilometer | Independently=0 | Need some help=0.5 | Unable=1 |  |
| Lift a weight of 5 kg | Independently=0 | Need some help=0.5 | Unable=1 |  |
| Continuously crouch and stand up three times | Independently=0 | Need some help=0.5 | Unable=1 |  |
| Use public transportation | Independently=0 | Need some help=0.5 | Unable=1 |  |
| Needs assistance bathing | No need help=0 | Need some help=0.5 | Need help completely=1 |  |
| Needs assistance dressing | No need help=0 | Need some help=0.5 | Need help completely=1 |  |
| Needs assistance toileting | No need help=0 | Need some help=0.5 | Need help completely=1 |  |
| Needs assistance in indoor transferring | No need help=0 | Need some help=0.5 | Need help completely=1 |  |
| Needs assistance eating | No need help=0 | Need some help=0.5 | Need help completely=1 |  |
| Incontinence | No need help=0 | Need some help=0.5 | Need help completely=1 |  |
| Based on the mini mental state examination* | MMSE scores≥18=0 | MMSE scores<18=1 |  |  |
| Put hand behind neck | Neither=0 | Only one hand=0.5 | Both hands=1 |  |
| Put hand behind lower back | Neither=0 | Only one hand=0.5 | Both hands=1 |  |
| Raise arm upright | Neither=0 | Only one hand=0.5 | Both hands=1 |  |
| Stand up from sitting in a chair | Without using hands=0 | Using hands=0.5 | Cannot=1 |  |
| Pick up a book from the floor | Yes, standing=0 | Yes, sitting=0.5 | Cannot=1 |  |
| Self-rated health | Very good/Good=0 | So so=0.5 | Bad=0.75 | Very bad=1 |
| Health status compared with last year | Much /Slightly better=0 | Almost the same=0.5 | Slightly worse=0.75 | Much worse=1 |
| Interviewer-rated health | Surprisingly healthy=0 | Relatively healthy=0.5 | Moderately ill=0.75 | Very ill=1 |
| Suffering from hypertension | No=0 | Yes=1 |  |  |
| Suffering from diabetes | No=0 | Yes=1 |  |  |
| Suffering from heart disease | No=0 | Yes=1 |  |  |
| Suffering from stroke, CVD | No=0 | Yes=1 |  |  |
| Suffering from pulmonary disease | No=0 | Yes=1 |  |  |
| Suffering from pulmonary tuberculosis | No=0 | Yes=1 |  |  |
| Suffering from cataracts | No=0 | Yes=1 |  |  |
| Suffering from glaucoma | No=0 | Yes=1 |  |  |
| Suffering from cancer | No=0 | Yes=1 |  |  |
| Suffering from prostate tumor | No=0 | Yes=1 |  |  |
| Suffering from gastric or duodenal ulcer | No=0 | Yes=1 |  |  |
| Suffering from Parkinson’s disease | No=0 | Yes=1 |  |  |
| Suffering from bedsore | No=0 | Yes=1 |  |  |
| Suffering from arthritis | No=0 | Yes=1 |  |  |
| Suffering from dementia | No=0 | Yes=1 |  |  |

***Note:*** CVD (cerebrovascular disease); MMSE score cut off =18 [11] (Zeng, Gu, Purser, Hoenig, & Christakis, 2010)

**Table S2** Measurement of variables in 2002

| **Variables** | **Measurement** |
| --- | --- |
| **Sociodemographic Characteristics** |  |
| Age | Continuous Variable |
| Sex | 0=Male, 1=Female |
| Ethnicity | 0=Han Ancestry, 1=Minority |
| Education | 0= Literate, 1=Illiterate |
| Marital Status | 0=Unmarried/Separated/Divorced/Widowed, 1=Married |
| Residence | 0=City, 1=Town, 2=Rural |
| Co-residence | 0=Living Alone, 1=Living with Household Members |
| Primary Occupation | 0=labor work,1=Mental work |
| Be Retired | 0=Yes, 1=No |
| Economic Status | 0=Low level, 1=Average level,  2=High level |
| Medical Service in Childhood | 0=Inadequate, 1=Adequate |
| Childhood starvation | 0=No, 1=Yes |
| **Lifestyles** |  |
| Regular Fruit Intake | 0=Low Frequency,  1=High Frequency |
| Regular Vegetables Intake | 0=Low Frequency,  1=High Frequency |
| Current Smoker | 0=No, 1=Yes |
| Ex-smoker | 0=No, 1=Yes |
| Current Drinker | 0=No, 1=Yes |
| Ex-drinker | 0=No, 1=Yes |
| Current Exerciser | 0=No, 1=Yes |
| Ex-Exerciser | 0=No, 1=Yes |
| Regular Physical Labor | 0=No, 1=Yes |
| Housework | 0=High Frequency, 1=Low Frequency |
| Outdoor Activities | 0=High Frequency, 1=Low Frequency |
| Garden Work | 0=High Frequency, 1=Low Frequency |
| Read Newspapers/Books | 0=High Frequency, 1=Low Frequency |
| Raise Domestic Animals/Pets | 0=High Frequency, 1=Low Frequency |
| Play Cards/Mah-Jong | 0=High Frequency, 1=Low Frequency |
| Watch TV Or Listen Radio | 0=High Frequency, 1=Low Frequency |
| Organized activities | 0=High Frequency, 1=Low Frequency |
| Religious Activities | 0=High Frequency, 1=Low Frequency |
| **Mental State & Physical Activity** |  |
| MMSE Score* | Continuous Variable |
| IADL Score* | Continuous Variable |
| ADL Score* | Continuous Variable |
| PWB * | Continuous Variable |
| **Chronic Diseases** | 0=Low level, 1=High level |
| Hypertension | 0=No, 1=Yes |
| Diabetes | 0=No, 1=Yes |
| Heart Disease | 0=No, 1=Yes |
| Stroke/CVD* | 0=No, 1=Yes |
| Bronchitis, Emphysema, Asthma, Pneumonia | 0=No, 1=Yes |
| Pulmonary tuberculosis | 0=No, 1=Yes |
| Cataract | 0=No, 1=Yes |
| Glaucoma | 0=No, 1=Yes |
| Cancer | 0=No, 1=Yes |
| Prostate tumor | 0=No, 1=Yes |
| Gastric/duodenal ulcer | 0=No, 1=Yes |
| Parkinson’s disease | 0=No, 1=Yes |
| Bedsore | 0=No, 1=Yes |
| Arthritis | 0=No, 1=Yes |
| Dementia | 0=No, 1=Yes |
| **Objective Examination** |  |
| Weight | Continuous Variable |
| Length From Wrist to Shoulder | Continuous Variable |
| Length From Kneel to Floor | Continuous Variable |
| Systolic | Continuous Variable |
| Diastolic | Continuous Variable |
| Heart Rate | Continuous Variable |
| **Present Medical Service** |  |
| Medical Costs Payer | 0=Public medical care fund, 1=Self, 2=Family and children, 3=Others |

*CVD**=**cardiovascular disease;

*PWB =Psychological well-being; *MMSE=Mini-Mental State Examination;

*IADL= Instrumental activity of daily living; *ADL= Basic activity of daily living

**Table S3** Performance of group-based trajectory model

| Fit statistic |  | Number of classes | | | | |
| --- | --- | --- | --- | --- | --- | --- |
|  | 1 | 2 | 3 | 4 | 5 | 6 |
| BIC* | 12897.51 | 14639.21 | 15165.02 | 15503.45 | 15650.04 | 15753.73 |
| AIC* | 12910.07 | 14664.47 | 15202.91 | 15553.96 | 15713.18 | 15829.50 |
| Class proportion | Class1, 100.00% | Class 1, 82.54% | Class 1, 7.44% | Class 1, 37.63% | Class 1, 5.85% | Class 1, 4.07% |
|  |  | Class 2, 17.46% | Class 2, 59.90% | Class 2, 15.61% | Class 2, 1.67% | Class 2, 2.05% |
|  |  |  | Class 3, 32.66% | Class 3, 43.88% | Class 3, 40.86% | Class 3, 42.49% |
|  |  |  |  | Class 4, 2.88% | Class 4, 36.48% | Class 4, 14.90% |
|  |  |  |  |  | Class 5, 15.14% | Class 5, 1.49% |
|  |  |  |  |  |  | Class 6, 35.00% |
| APP | Class1, 1.00 | Class 1, 0.81 | Class 1, 0.88 | Class 1, 0.73 | Class 1, 0.81 | Class 1, 0.80 |
|  |  | Class 2, 0.88 | Class 2, 0.84 | Class 2, 0.81 | Class 2, 0.88 | Class 2, 0.90 |
|  |  |  | Class 3, 0.79 | Class 3, 0.70 | Class 3, 0.67 | Class 3, 0.69 |
|  |  |  |  | Class 4, 0.92 | Class 4, 0.71 | Class 4, 0.75 |
|  |  |  |  |  | Class 5, 0.74 | Class 5, 0.82 |
|  |  |  |  |  |  | Class 6, 0.71 |

BIC*: Bayesian information criteria; AIC*: Akaike’s information criterion

Class proportion: Designated that no less than 5% of total count in a class

APP: average posterior probabilities (preferably > 0.70 in a class)

**Table S4** The parameters of final two-group of frailty trajectory

| Trajectory group | Parameter | Maximum likelihood estimates | | | |
| --- | --- | --- | --- | --- | --- |
|  |  | Est. | SE | z value | p value |
| Class 1: stable-growth (n = 3,370, 82.54%) | Intercept | 1.02 | 0.07 | 14.203 | 0.000 |
|  | Linear (age) | -0.03 | 0.00 | -15.822 | 0.000 |
|  | Quadratic (age^2^) | <0.01^*^ | <0.01^**^ | 19.016 | 0.000 |
| Class 2: rapid-growth (n = 713, 17.46%) | Intercept | -0.67 | 0.21 | -3.233 | 0.001 |
|  | Linear (age) | 0.01 | 0.01 | 1.936 | 0.053 |
|  | Quadratic (age^2^) | <0.01^#^ | <0.01^##^ | 0.507 | 0.612 |
| Est.: parameter estimate, SE: standard error of parameter estimate | | | | | |

<0.01^*^：0.00020，<0.01^**^：0.00001

<0.01^#^：0.00002，<0.01^##^：0.00003

**Table S5** Selected characteristics comparison between analytical sample and drop-out sample among those aged 65 years or older in 2002

|  | **Total sample (N=16,315)** | **Trajectory Class** | | **P-value#** |
| --- | --- | --- | --- | --- |
|  |  | **drop-out subjects  n = (12,232)** | **analytical subjects**  **n = (4,083)** |  |
| **Marital Status** |  |  |  |  |
| Unmarried/Separated/Divorced/Widowed | 4887 (30.0%) | 2884 (23.6%) | 2003 (49.1%) | **0.000** |
| Married | 11428 (70.0%) | 9348 (76.4%) | 2080 (50.9%) |  |
| **Alcohol consumption** |  |  |  |  |
| No | 11241 (68.9%) | 8445 (69.0%) | 2794 (68.4%) | **0.000** |
| Yes | 5074 (31.1%) | 3787 (31.0%) | 1289 (31.6%) |  |
| **Arthritis** |  |  |  |  |
| No | 13561 (83.1%) | 10263 (83.9%) | 3298 (80.8%) | **0.000** |
| Yes | 2754 (16.9%) | 1969 (16.1%) | 785 (19.2%) |  |
| **Ex-Smoker** |  |  |  |  |
| No | 10835 (66.4%) | 8236 (67.3%) | 2599 (63.7%) | **0.000** |
| Yes | 5480 (33.6%) | 3996 (32.7%) | 1484 (36.3%) |  |
| **Stroke, cerebrovascular disease** |  |  |  |  |
| No | 15459 (94.8%) | 11555 (94.5%) | 3904 (95.6%) | **0.004** |
| Yes | 856 (5.2%) | 677 (5.5%) | 179 (4.4%) |  |
| **Ex-Exerciser** |  |  |  |  |
| No | 10226 (62.7%) | 7712 (63.0%) | 2514 (61.6%) | 0.091 |
| Yes | 6089 (37.3%) | 4520 (37.0%) | 1569 (38.4%) |  |
| **Medical Costs Payer** |  |  |  |  |
| Public medical care fund | 2125 (13.0%) | 1564 (12.8%) | 561 (13.7%) | **0.000** |
| Self | 1958 (12.0%) | 1180 (9.6%) | 778 (19.1%) |  |
| Family and children | 11832 (72.5%) | 9154 (74.9%) | 2678 (65.6%) |  |
| Others | 400 (2.5%) | 334 (2.7%) | 66 (1.6%) |  |
| **Regular Fruit Intake** |  |  |  |  |
| Low Frequency | 10849 (66.5%) | 8166 (66.8%) | 2683 (65.7%) | 0.219 |
| High Frequency | 5466 (33.5%) | 4066 (33.2%) | 1400 (34.3%) |  |
| **Residence** |  |  |  |  |
| City | 3898 (23.9%) | 3081 (25.2%) | 817 (20.0%) | **0.000** |
| Town | 3619 (22.2%) | 2726 (22.3%) | 893 (21.9%) |  |
| Rural | 8798 (53.9%) | 6425 (52.5%) | 2373 (58.1%) |  |
| **Economic Status** |  |  |  |  |
| Low level | 2613 (16.0%) | 2022 (16.5%) | 591 (14.5%) | **0.008** |
| Average level | 10887 (66.7%) | 8110 (66.3%) | 2777 (68.0%) |  |
| High level | 2815 (17.3%) | 2100 (17.2%) | 715 (17.5%) |  |
| **Heart disease** |  |  |  |  |
| No | 14877 (91.2%) | 11162 (91.3%) | 3715 (91.0%) | 0.604 |
| Yes | 1438 (8.8%) | 1070 (8.7%) | 368 (9.0%) |  |
| **Hypertension** |  |  |  |  |
| No | 13802 (84.6%) | 10428 (85.3%) | 3374 (82.6%) | **0.000** |
| Yes | 2513 (15.4%) | 1804 (14.7%) | 709 (17.4%) |  |
| **Raise domestic animals** |  |  |  |  |
| Low Frequency | 12863 (78.8%) | 10289 (84.1%) | 2574 (63.0%) | **0.000** |
| High Frequency | 3452 (21.2%) | 1943 (15.9%) | 1509 (37.0%) |  |
| **Childhood starvation** |  |  |  |  |
| No | 5728 (35.1%) | 4343 (35.5%) | 1385 (33.9%) | 0.066 |
| Yes | 10587 (64.9%) | 7889 (64.5%) | 2698 (66.1%) |  |
| **Medical Service in Childhood** |  |  |  |  |
| Inadequate | 9593 (58.8%) | 7225 (59.1%) | 2368 (58.0%) | **0.029** |
| Adequate | 6722 (41.2%) | 5007 (40.9%) | 1715 (42.0%) |  |
| **Watch TV and/or listen to radio** |  |  |  |  |
| Low Frequency | 7612 (46.7%) | 6373 (52.1%) | 1239 (30.3%) | **0.000** |
| High Frequency | 8703 (53.3%) | 5859 (47.9%) | 2844 (69.7%) |  |
| **Education** |  |  |  |  |
| Literate | 10085 (61.8%) | 7888 (64.5%) | 2197 (53.8%) | **0.000** |
| Illiterate | 6230 (38.2%) | 4344 (35.5%) | 1886 (46.2%) |  |
| **Systolic** | 134.100±17.590 | 134.321±0.159 | 133.436±0.277 | **0.005** |
| **Diastolic** | 85.376±12.246 | 85.582±0.110 | 84.758±0.196 | **0.000** |
| **Heart rate** | 72.727±8.275 | 72.824±0.077 | 72.437±0.117 | **0.010** |
| **Weight (kg)** | 49.012±10.814 | 48.186±0.098 | 51.487±0.160 | **0.000** |
| **Length from wrist to shoulder** | 49.455±5.733 | 49.273±0.052 | 50.002±0.087 | **0.000** |
| **Right knee to the floor (CM)** | 46.082±5.488 | 45.843±0.050 | 46.797±0.085 | **0.000** |
| **ADL** | 10.835±2.411 | 10.513±0.024 | 11.799±0.014 | **0.000** |
| **IADL** | 9.922±6.049 | 8.555±0.055 | 14.018±0.055 | **0.000** |
| **PWB** | 26.279±4.212 | 26.127±0.038 | 26.736±0.063 | **0.000** |
| **MMSE** | 24.992±5.049 | 24.248±0.048 | 27.221±0.052 | **0.000** |

***NOTE*:** Values are presented as mean ± standard deviation, number (%).

*ADL (basic activity of daily living)

* IADL (instrumental activity of daily living); *MMSE (Mini-Mental State Examination)

# ANOVA test and chi square test were performed, and the null hypothesis is no difference across the two groups

**Table S6** Selected characteristics comparison between trajectories classes in 2002

|  | **Total sample (N=4,083)** | **Trajectory Class** | | **P-value#** |
| --- | --- | --- | --- | --- |
|  |  | **stable-growth** | **rapid-growth** |  |
|  |  | **n = (3,370)** | **n = (713)** |  |
| **Marital Status** |  |  |  |  |
| Unmarried/Separated/Divorced/Widowed | 2003 (49.1%) | 1635 (48.5%) | 368 (51.6%) | **0.013** |
| Married | 2080 (50.9%) | 1735 (51.5%) | 345 (48.4%) |  |
| **Alcohol consumption** |  |  |  |  |
| No | 2794 (68.4%) | 2283 (67.7%) | 511 (71.7%) | **0.041** |
| Yes | 1289 (31.6%) | 1087 (32.3%) | 202 (28.3%) |  |
| **Arthritis** |  |  |  |  |
| No | 3298 (80.8%) | 2763 (82.0%) | 535 (75.0%) | **0.000** |
| Yes | 785 (19.2%) | 607 (18.0%) | 178 (25.0%) |  |
| **Ex-Smoker** |  |  |  |  |
| **No** | 2599 (63.7%) | 2132 (63.3%) | 467 (65.5%) | 0.260 |
| Yes | 1484 (36.3%) | 1238 (36.7%) | 246 (34.5%) |  |
| **Stroke, cerebrovascular disease** |  |  |  |  |
| No | 3904 (95.6%) | 3282 (97.4%) | 622 (87.2%) | **0.000** |
| Yes | 179 (4.4%) | 88 (2.6%) | 91 (12.8%) |  |
| **Ex-Exerciser** |  |  |  |  |
| No | 2514 (61.6%) | 2080 (61.7%) | 434 (60.9%) | 0.671 |
| Yes | 1569 (38.4%) | 1290 (38.3%) | 279 (39.1%) |  |
| **Medical Costs Payer** |  |  |  |  |
| Public medical care fund | 561 (13.7%) | 446 (13.2%) | 115 (16.1%) | **0.039** |
| Self | 778 (19.1%) | 640 (19.0%) | 138 (19.4%) |  |
| Family and children | 2678 (65.6%) | 2235 (66.3%) | 443 (62.1%) |  |
| Others | 66 (1.6%) | 49 (1.5%) | 17 (2.4%) |  |
| **Regular Fruit Intake** |  |  |  |  |
| Low Frequency | 2683 (65.7%) | 2218 (65.8%) | 465 (65.2%) | 0.760 |
| High Frequency | 1400 (34.3%) | 1152 (34.2%) | 248 (34.8%) |  |
| **Residence** |  |  |  |  |
| city | 817 (20.0%) | 634 (18.8%) | 183 (25.7%) | **0.000** |
| town | 893 (21.9%) | 736 (21.9%) | 157 (22.0%) |  |
| rural | 2373 (58.1%) | 2000 (59.3%) | 373 (52.3%) |  |
| **Economic Status** |  |  |  |  |
| Low level | 591 (14.5%) | 475 (14.1%) | 116 (16.3%) | 0.315 |
| Average level | 2777 (68.0%) | 2300 (68.2%) | 477 (66.9%) |  |
| High level | 715 (17.5%) | 595 (17.7%) | 120 (16.8%) |  |
| **Heart disease** |  |  |  |  |
| No | 3715 (91.0%) | 3137 (93.1%) | 578 (81.1%) | **0.000** |
| Yes | 368 (9.0%) | 233 (6.9%) | 135 (18.9%) |  |
| **Hypertension** |  |  |  |  |
| No | 3374 (82.6%) | 2877 (85.4%) | 497 (69.7%) | **0.000** |
| Yes | 709 (17.4%) | 493 (14.6%) | 216 (30.3%) |  |
| **Raise domestic animals** |  |  |  |  |
| Low Frequency | 2574 (63.0%) | 2070 (61.4%) | 504 (70.7%) | **0.000** |
| High Frequency | 1509 (37.0%) | 1300 (38.6%) | 209 (29.3%) |  |
| **Childhood starvation** |  |  |  |  |
| No | 1385 (33.9%) | 1146 (34.0%) | 239 (33.5%) | 0.804 |
| Yes | 2698 (66.1%) | 2224 (66.0%) | 474 (66.5%) |  |
| **Medical Service in Childhood** |  |  |  |  |
| Inadequate | 2368 (58.0%) | 1952 (57.9%) | 416 (58.3%) | 0.836 |
| Adequate | 1715 (42.0%) | 1418 (42.1%) | 297 (41.7%) |  |
| **Watch TV and/or listen to radio** |  |  |  |  |
| Low Frequency | 1239 (30.3%) | 1010 (30.0%) | 229 (32.1%) | 0.257 |
| High Frequency | 2844 (69.7%) | 2360 (70.0%) | 484 (67.9%) |  |
| **Education** |  |  |  |  |
| Literate | 2197 (53.8%) | 1774 (52.6%) | 423 (59.3%) | **0.001** |
| Illiterate | 1886 (46.2%) | 1596 (47.4%) | 290 (40.7%) |  |
| **Systolic** | 133.440±17.704 | 132.850±17.199 | 136.190±19.700 | **0.000** |
| **Diastolic** | 84.760±12.500 | 84.530±12.385 | 85.840±12.982 | **0.011** |
| **Heart rate** | 72.440±7.486 | 72.380±7.436 | 72.720±7.717 | 0.261 |
| **Weight ( kg）** | 51.490±10.216 | 51.260±10.032 | 52.580±10.987 | **0.002** |
| **Length from wrist to shoulder** | 50.000±5.573 | 50.130±5.564 | 49.400±5.577 | **0.001** |
| **Right knee to the floor (CM)** | 46.800±5.431 | 46.840±5.408 | 46.580±5.537 | 0.248 |
| **ADL** | 11.799±0.901 | 11.896±0.517 | 26.764±3.634 | **0.000** |
| **IADL** | 14.018±3.514 | 14.431±2.925 | 12.065±5.068 | **0.000** |
| **PWB** | 26.736±4.050 | 26.852±4.040 | 26.192±4.056 | **0.000** |
| **MMSE** | 27.221±3.342 | 27.317±3.269 | 26.764±3.634 | **0.000** |

***NOTE*:** Values are presented as mean ± standard deviation, number (%).

*ADL (basic activity of daily living)

* IADL (instrumental activity of daily living); *MMSE (Mini-Mental State Examination)

# ANOVA test and chi square test were performed, and the null hypothesis is no difference across the two classes

**Tables S7** Comparison of sample characteristics by multivariate logistic model between stable-growth and rapid-growth frailty trajectories, the stable-growth was set as the reference. The numbers of stable-growth and rapid-growth frailty trajectories were 3,370 and 713 respectively

|  | **Multivariate Logistic** | |
| --- | --- | --- |
|  | OR | *P* |
| **Marital Status (Ref: Unmarried/Separated/Divorced/Widowed)** | |  |
| Married | 0.693 (0.571-0.840) | **0.000** |
| **Alcohol consumption (Ref: No)** |  |  |
| Yes | 0.878 (0.710-1.085) | 0.228 |
| **Arthritis (Ref: No)** |  |  |
| Yes | 1.314 (1.063-1.623) | **0.011** |
| **Ex-Smoker (Ref: No)** |  |  |
| Yes | 1.070 (0.867-1.320) | 0.528 |
| **Stroke, cerebrovascular disease (Ref: No)** |  |  |
| Yes | 3.041 (2.139-4.323) | **0.000** |
| **Ex-Exerciser (Ref: No)** |  |  |
| Yes | 0.978 (0.804-1.190) | 0.825 |
| **Medical Costs Payer (Ref: Public medical care fund)** |  |  |
| Self | 0.729 (0.368-1.443) | 0.364 |
| Family and children | 0.782 (0.405-1.511) | 0.465 |
| Others | 0.598 (0.316-1.129) | 0.113 |
| **Regular Fruit Intake (Ref: Low Frequency)** |  |  |
| High Frequency | 0.940 (0.770-1.147) | 0.542 |
| **Residence (Ref: City)** |  |  |
| Town | 1.085 (0.830-1.417) | 0.552 |
| Rural | 1.031 (0.820-1.296) | 0.796 |
| **Economic Status (Ref: Poor)** |  |  |
| Average | 0.994 (0.712-1.388) | 0.973 |
| Rich | 1.010 (0.791-1.290) | 0.935 |
| **Heart disease (Ref: No)** |  |  |
| Yes | 2.090 (1.606-2.720) | **0.000** |
| **Hypertension (Ref: No)** |  |  |
| Yes | 1.846 (1.449-2.351) | **0.000** |
| **Raise domestic animals (Ref: Low Frequency)** |  |  |
| High Frequency | 0.966 (0.784-1.191) | 0.749 |
| **Childhood starvation (Ref: No)** |  |  |
| Yes | 1.070 (0.871-1.315) | 0.517 |
| **Medical Service in Childhood (Ref: Inadequate)** |  |  |
| Adequate | 1.010 (0.831-1.228) | 0.920 |
| **Watch TV and/or listen to radio (Ref: Low Frequency)** | |  |
| High Frequency | 1.205 (0.972-1.493) | 0.089 |
| **Education (Ref: Literate)** |  |  |
| Illiterate | 0.643 (0.523-0.791) | **0.000** |
| **Systolic** | 1.000 (0.993-1.006) | 0.877 |
| **Diastolic** | 0.996 (0.988-1.005) | 0.377 |
| **Heart rate** | 1.005 (0.994-1.017) | 0.395 |
| **Weight (kg)** | 1.020 (1.010-1.031) | **0.000** |
| **Length from wrist to shoulder** | 0.972 (0.949-0.996) | **0.021** |
| **Right knee to the floor (CM)** | 1.007 (0.983-1.033) | 0.553 |
| **ADL** | 0.827 (0.740-0.925) | **0.001** |
| **IADL** | 0.875 (0.849-0.902) | **0.000** |
| **PWB** | 0.978 (0.955-1.002) | 0.070 |
| **MMSE** | 1.024 (0.995-1.054) | 0.107 |

***Note:*** OR = odds ratio; Ref = reference;

*PWB = Psychological well-being; *MMSE = Mini-Mental State Examination;

*ADL = Basic activity of daily living; * IADL=instrumental activity of daily living


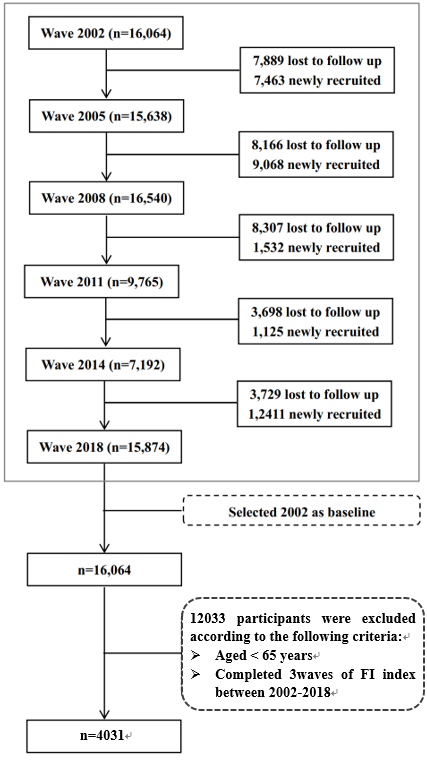

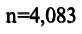

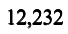

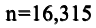


**Figure S1** The follow-up information of CLHLS-HF database and the sample selection


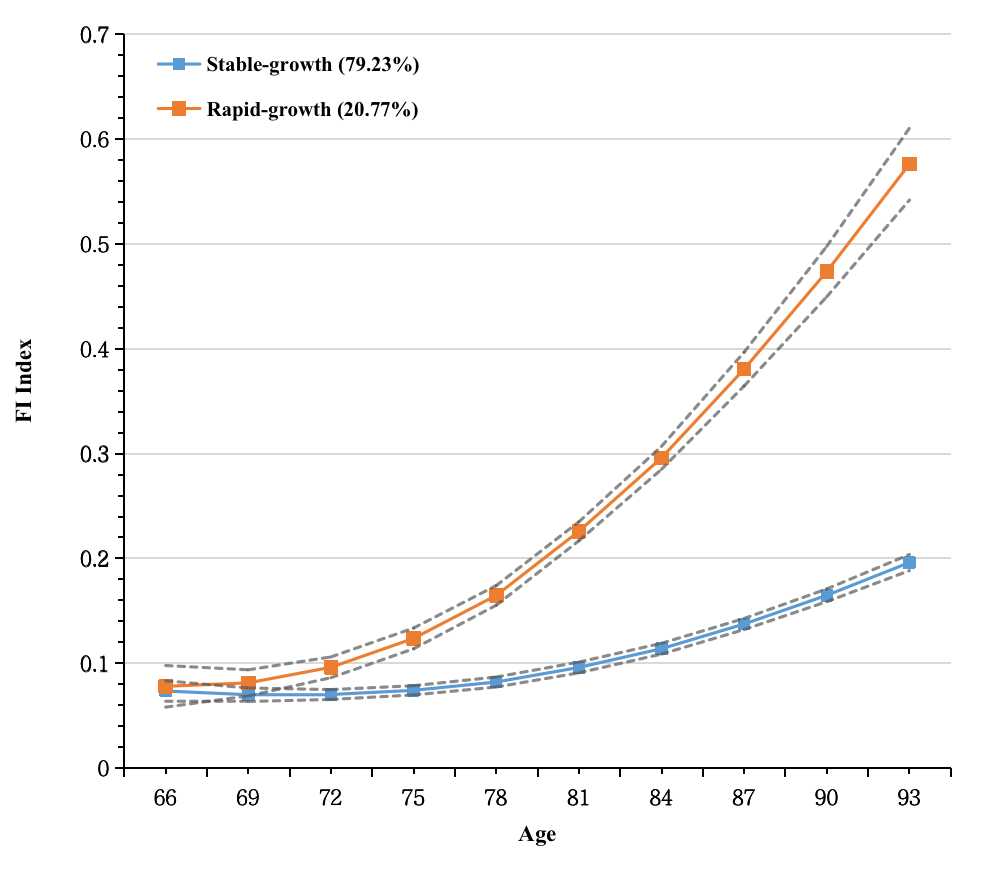


**Figure S2** Heterogenous frailty trajectories classes of older adults for all six waves (n= 617). FI index was generated on the basis of the number of deficits present in an individual (a range of symptoms, sensory deficits, clinical signs, diseases, disabilities and abnormal laboratory test results) divided by the total number of deficits possible, ranging from 0–1. Two-class was selected as final model based on GBTM (Bayesian information criteria=4, 154.58; Akaike information criterion=4,172.28), including “stable-growth” class, “rapid-growth” class, similar to our analytic sample in main text (n= 4,083). The solid lines represent the means, and the dashed lines represent the 95% confidence intervals of the mean.


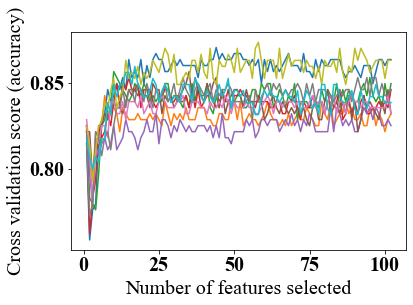


**Figure S3** Feature selection based on recursive feature elimination with ten-fold cross-validation (base model: random forest)

**References**

1. Yin S, Yang Q, Xiong J, Li T, Zhu X: **Social Support and the Incidence of Cognitive Impairment Among Older Adults in China: Findings From the Chinese Longitudinal Healthy Longevity Survey Study**. *Front Psychiatry* 2020, **11**:254.

2. Chen L, Lu B: **Cognitive reserve regulates the association between hearing difficulties and incident cognitive impairment evidence from a longitudinal study in China**. *International Psychogeriatrics* 2020, **32**(5):635-643.

3. Yi Z, Vaupel J, Zhenyu X, Chunyuan Z, Yuzhi L: **The Healthy Longevity Survey and the Active Life Expectancy of the Oldest Old in China**. *Population: An English Selection* 2001, **13**:95-116.

4. Gu D, Zeng Y: **Sociodemographic Effects on the Onset and Recovery of ADL Disability among Chinese Oldest-old**. *Demographic Research* 2004, **11**:1-42.

5. Lawton Mp Fau - Brody EM, Brody EM: **Assessment of older people: self-maintaining and instrumental activities of daily living**. (0016-9013 (Print)).

6. Zhang L, Bi X, Ding Z: **Health lifestyles and Chinese oldest-old's subjective well-being-evidence from a latent class analysis**. *BMC Geriatr* 2021, **21**(1):206.

7. Zhou Z, Cai L, Zhuang M, Hong YA, Fang Y: **A longitudinal analysis of the association between the living arrangements and psychological well-being of older Chinese adults: the role of income sources**. *BMC Geriatr* 2019, **19**(1):347.

8. Folstein MF, Folstein SE, McHugh PR: **"Mini-mental state". A practical method for grading the cognitive state of patients for the clinician**. *J Psychiatr Res* 1975, **12**(3):189-198.

9. Zeng Y, Feng Q, Hesketh T, Christensen K, Vaupel JW: **Survival, disabilities in activities of daily living, and physical and cognitive functioning among the oldest-old in China: a cohort study**. *Lancet* 2017, **389**(10079):1619-1629.

10. Goksuluk D, Zararsiz G, Korkmaz S, Eldem V, Zararsiz GE, Ozcetin E, Ozturk A, Karaagaoglu AE: **MLSeq: Machine learning interface for RNA-sequencing data**. *Computer Methods and Programs in Biomedicine* 2019, **175**:223-231.

11. Zeng Y, Gu D, Purser J, Hoenig H, Christakis N: **Associations of environmental factors with elderly health and mortality in China**. *Am J Public Health* 2010, **100**(2):298-305.
